# T Wave Safety Margin during the Process of ICD Implantation As a Novel Predictor of T Wave Oversensing

**DOI:** 10.3389/fphys.2017.00659

**Published:** 2017-09-01

**Authors:** Ya-Xun Sun, Jing Gao, Chen-Yang Jiang, Yu-Mei Xue, Yi-Zhou Xu, Gang Liu, Ji-Hong Guo, Xia Sheng, Yang Ye, Hong He, Yun-Tao Zhao, Hector Barajas-Martinez, Guo-Sheng Fu, Dan Hu

**Affiliations:** ^1^Department of Cardiology, Sir Run Run Shaw Hospital, Zhejiang University School of Medicine Hangzhou, China; ^2^Department of Cardiology, Guangdong Cardiovascular Institute, Guangdong General Hospital Guangzhou, China; ^3^Department of Cardiology, Hangzhou First People's Hospital Hangzhou, China; ^4^Department of Cardiology, First Hospital of Hebei Medical University Shijiazhuang, China; ^5^Division of Cardiology, Peking University People's Hospital Beijing, China; ^6^Department of Cardiology, Aerospace Center Hospital Beijing, China; ^7^Masonic Medical Research Laboratory Utica, NY, United States; ^8^Department of Cardiology and Cardiovascular Research Institute, Renmin Hospital of Wuhan University Wuhan, China

**Keywords:** implantable cardioverter-defibrillator, T wave oversensing, safety margin, inappropriate therapy, intracardiac electrocardiogram

## Abstract

**Introduction:** T wave oversensing (TWOS) is a major drawback of implantable cardioverter defibrillator (ICD) and data on predictors of TWOS in ICD is limited. We aimed to calculate a novel index of T wave safety margin (TWSM) and assess its potential for evaluating TWOS during the procedure of ICD implantation.

**Methods and Results:** Thirty-two consecutive patients with ICD implantation were enrolled. During each procedure of ICD implantation, different ICD generators were connected to implanted sensing lead through active-fixation leads and bridging cables. R and T wave amplitudes were measured on ICD printouts according to the gain. The ICDs were programed to the most sensitive settings to reveal possible TWOS. A novel index TWSM was calculated according to the corresponding sensing algorithm of ICD. There was discrepancy of R wave amplitudes measured by different ICDs (*P* < 0.01). In Fortify and Teligen ICDs, T wave amplitudes showed no difference (*P* > 0.05) and TWSMs were sufficiently high (post sensing: 13.0 ± 7.6 and 28.3 ± 16.5, respectively, post pacing: 5.0 ± 2.2 and 4.6 ± 0.9, respectively). In nine patients with 10 TWOS episodes detected during the procedure of ICD implantation, generators with the highest TWSM were chosen. Only one TWOS episode during pacing was recorded during the 25 ± 7 mo follow-up period.

**Conclusions:** We first propose the index of TWSM during ICD implantation as a potentially efficient predictor for TWOS. Evaluation of TWSM might help to reduce TWOS episodes in patients with high risk of TWOS. Prospective studies are warranted to validate this index and its potential to reduce TWOS episodes.

## Introduction

Implantable cardioverter-defibrillators (ICDs), either alone or with cardiac resynchronization therapy (CRT), reduce mortality in patients at high risk for cardiac death (Moss et al., 1996, 2002, 2009). However, inappropriate ICD therapy can result in impaired quality of life, psychiatric disturbance, and life-threatening ventricular arrhythmias (Mark et al., 2008; Juan and Pollack, 2010; van Rees et al., 2011; Powell et al., 2013). How to reduce inappropriate therapy is a significant challenge in ICD patients.

T wave oversensing (TWOS) is one of the major reasons of inappropriate ICD therapy. In some subgroups of patients, such as those with Brugada syndrome, long QT syndrome, short QT syndrome, hypertrophic cardiomyopathy, and younger patients (Schimpf et al., 2003; Weretka et al., 2003; Sacher et al., 2006, 2013; Magnusson et al., 2015; Olde Nordkamp et al., 2016), TWOS after ICD implantation has been reported in 3 ~ 14% of these patients. However, the studies focusing on the predictors of TWOS in ICD are limited (Maesato et al., 2011). In these patients, evaluating the risk of TWOS during the procedure of ICD implantation might help in reducing TWOS episodes.

In this study, we introduced a novel TWSM index and evaluated its potential of reducing TWOS episodes in mid-term follow up.

## Methods

This study was approved by the Ethics Committee of the Sir Run Run Shaw Hospital at Zhejiang University. Informed written consents were obtained from all study participants.

### Clinical and device characteristics

From April 2013 to July 2014, 32 consecutive patients with ICD implantation indices were enrolled. The average age of the 32 patients (59.4% male) was 54.9 ± 12 y. The indications for ICD implantation and clinical characteristic of the patients are summarized in Table 1. The case with short QT syndrome was reported previously (Sun et al., 2010, 2011). Six ICDs (CRT/D) from three manufacturers (St. Jude Medical, Inc.; Medtronic, Inc.; and Boston Scientific, Inc.) were applied as testing ICDs in this study; all testing ICDs were functionally normal.

**Table 1:** Demographic and clinical characteristics of patients.

	**Patients (*n* = 32)**
Age, y/o (Mean ±*SD*)	54.9 ± 12.3
Male, *n* (%)	19 (59%)
Primary prevention, *n* (%)	23 (72%)
Secondary prevention, *n* (%)	9 (28%)
**INDICATIONS FOR ICD IMPLANTATION, *n* (%)**	
Dilated cardiomyopathy	10 (31%)
Reduced LVEF post-myocardial infarction	6 (19%)
Survived idiopathic cardiac arrest	5 (16%)
Brugada syndrome	4 (13%)
Hypertrophic cardiomyopathy	3 (9%)
Long-QT syndrome	2 (6%)
Alcoholic cardiomyopathy	1 (3%)
Short-QT syndrome	1 (3%)
LVEF, % (*n*)	48 ± 19 (32)
Dilated cardiomyopathy	31 ± 3(10)
Ischemic cardiomyopathy	33 ± 2 (6)
Hypertrophic cardiomyopathy	76 ± 5 (3)
Arrhythmias related	62 ± 10 (13)
Serum potassium (mmol/L)	3.9 ± 0.3

*ICD, Implantable cardioverter-defibrillator; LVEF, left ventricular ejection fraction*.

### *Ex vivo* ICD connection

**Figure 1:**
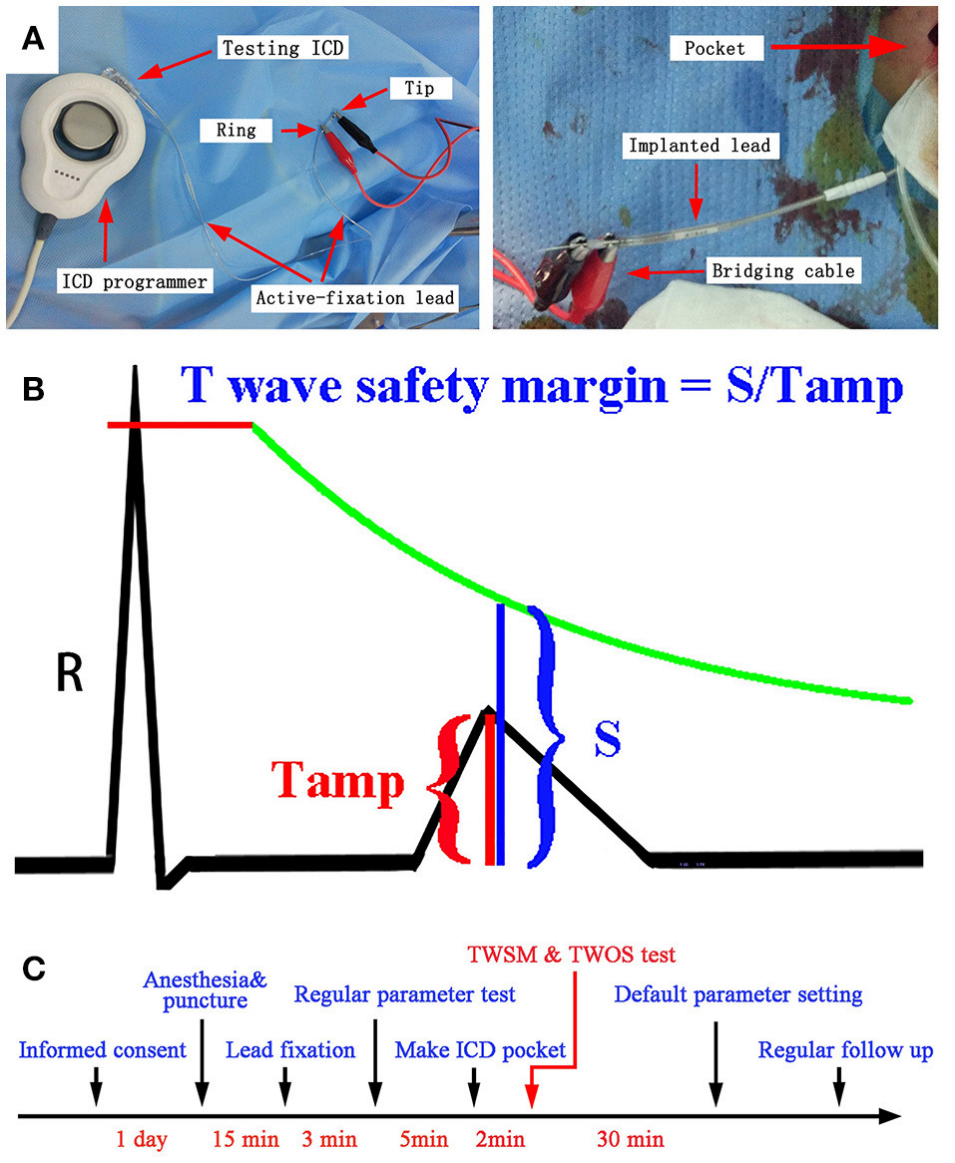
**(A)** *Ex vivo* ICD connection during the procedure of ICD implantation using the active-fixation lead. **(B)** TWSM measurements. The green line indicates the sensing threshold curve of ICD. **(C)** The timeline for the evaluation of TWSM.

*Ex vivo* connection was designed to enable evaluation of R and T wave amplitudes across all six testing ICDs in each patient. ICD implantation was performed under local anesthesia. All the shock leads were fixed in the lower ventricular septal. All the 32 defibrillation leads were dedicated bipolar leads. A bridging cable was connected to the connector pins of sensing lead by bipolar mode at 10 min after lead fixation to minimize the influence of acute injury current (Saxonhouse et al., 2005). According to the reported study and our experience, 10 min is long enough for normalizing acute injury current, and diminishing its influence on ST-T amplitude. The other end of the bridging cable was clipped onto both the tip and the ring electrode of an active-fixation lead (Guidant Fineline II EZ 4471). The active-fixation lead was then connected to the ventricular sensing channel of testing ICD. The testing ICD was programed by the corresponding ICD programmer (Figure 1A). The gain of the printed intracardiac electrocardiogram (IEGM) was set to an optimal value to avoid signal clipping and be eligible for T wave measurement.

After being connected to testing ICDs, ventricular IEGMs were printed by ICD programmers. The ventricular amplitude was measured by two methods: R wave sensing test by ICD programming, and signal measurement on ICD printouts. Three consecutive R waves were measured for an average value. T wave amplitudes post sensing and post pacing were measured by the same method (Figures 1A,B).

### TWOS and TWSM tests

After connected to the active-fixation lead, each testing ICD was programed to the most sensitive setting to reveal possible TWOS. TWOS episode was evaluated by two physicians with expertise in electrophysiology. If TWOS occurred, ventricular sensitivity was reduced and retested till TWOS was resolved. If TWOS occurred in certain type of ICD, another type of ICD generator with highest TWSM was implanted to avoid possible TWOS in the future. After the implantation, the ICD parameters were programed to the nominal setting unless changes were necessary. The timeline for TWSM is shown in Figure 1C. During each follow-up, the ICDs were temporarily programed to the most sensitive setting to reveal possible TWOS.

The TWSM was defined as the ratio of the ventricular sensing threshold at the T wave peak on IEGM to T wave amplitude (Figure 1B). The details to calculate TWSM in different ICDs were shown in Supplementary Table 1.

### Statistical analyses

All analyses were conducted using SPSS software (SPSS Inc., Chicago, IL, USA). Conformity to the normal distribution was evaluated for continuous variables with the Kolmogorov–Smirnov test. Comparisons between groups were made using a paired *t*-test, and One way repeated measure ANOVA was conducted to test paired data among three or more groups. R wave amplitudes measured by ICD programmers and on ICD printouts were compared using the Bland-Altman method as well as with linear regression and Pearson correlation analyses. Continuous variables are expressed as mean ± standard deviation. A two-tailed *P* < 0.05 was considered statistically significant.

## Results

### Basic characteristic

Patient characteristics are summarized in Table 1. Briefly, 59% of patients were men, with a mean age of 54.9 ± 12.3 years, mostly for primary prevention (72%) and with a mean serum potassium of 3.9 ± 0.3 mmol/L. Mean follow-up duration was 25 ± 7 months.

### R wave and T wave amplitudes

**Figure 2:**
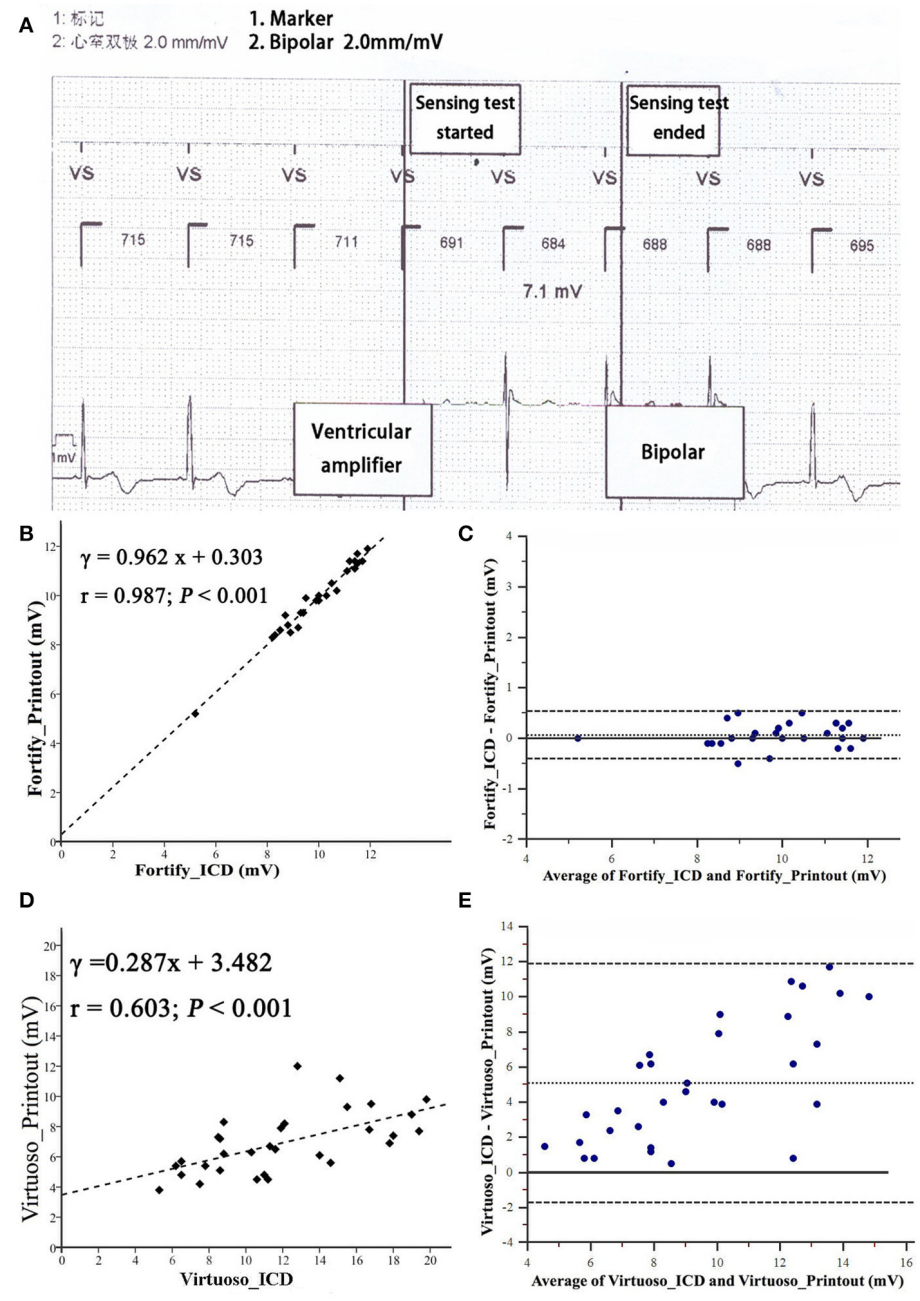
Bland-Altman analysis comparing R wave amplitudes measured from implantable cardioverter-defibrillator printouts. **(A)** Measurement of R wave amplitude by Fortify ICD. Noted that IEGM of ICD automatically switched to “Ventricular amplifier” channel to measure R wave amplitude. **(B,C)** R wave amplitude measured from by Fortify ICD. **(D,E)** R wave amplitude measured by Virtuoso ICD. Inner dashed line is the mean difference; outer dashed lines are mean ± 1.96 SD.

The R wave amplitude measured by ICD was consistent with that measured on “Ventricular Amplifier” channel in St. Jude ICDs and that on the “V” channel in the Teligen ICD, but was not consistent with “Pace/sense (bipolar)” channels of other four ICDs (Figure 2).

**Figure 3:**
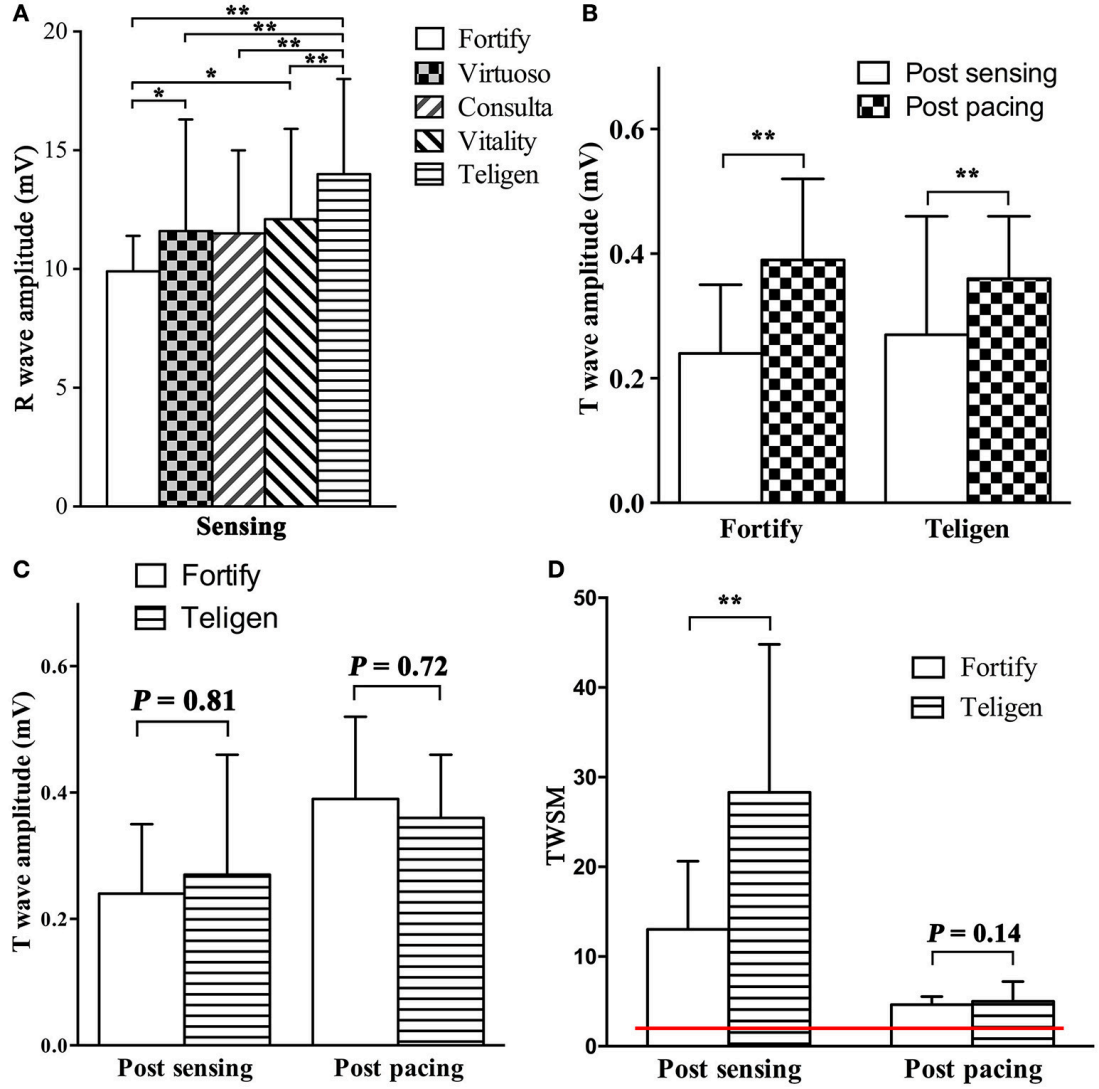
Comparison of R wave amplitude, T wave amplitudes and TWSMs by different ICDs. **(A)**. R wave amplitudes by different ICDs; **(B,C)** T wave amplitudes post sensing intrinsic rhythm and post pacing at 90/min in Fortify and Teligen ICDs. **(D)** TWSM after sensing intrinsic rhythm and pacing in Fortify and Teligen. Red line indicates safety margin of 2 (200%). ^*^P < 0.05; ^**^P < 0.01.

R wave amplitudes measured by five testing ICDs (Fortify, Virtuoso, Consulta, Vitality, and Teligen) were different (*P* < 0.01, *n* = 28, Figure 3A). Four patients were not included in R wave amplitude analysis, because their R wave amplitudes measured by Fortify ICDs exceeded 12 mV and could not be measured by ICD programmer accurately (only “>12 mV” was shown).

T wave amplitudes post pacing were higher than those post intrinsic rhythm (Fortify: 0.39 ± 0.13 vs. 0.24 ± 0.11 mV; *P* < 0.001, *n* = 21; Teligen: 0.36 ± 0.11 vs. 0.24 ± 0.10 mV; *P* < 0.01, *n* = 23. Figure 3B). There were no difference between T wave amplitudes measured by Fortify and Teligen ICDs (post-sensing: 0.26 ± 0.15 vs. 0.27 ± 0.19 mV; *P* = 0.81, *n* = 32; Post-pacing: 0.39 ± 0.11 vs. 0.38 ± 0.10 mV; *P* = 0.72, *n* = 19; Figure 3C).

### TWOS testing

**Figure 4:**
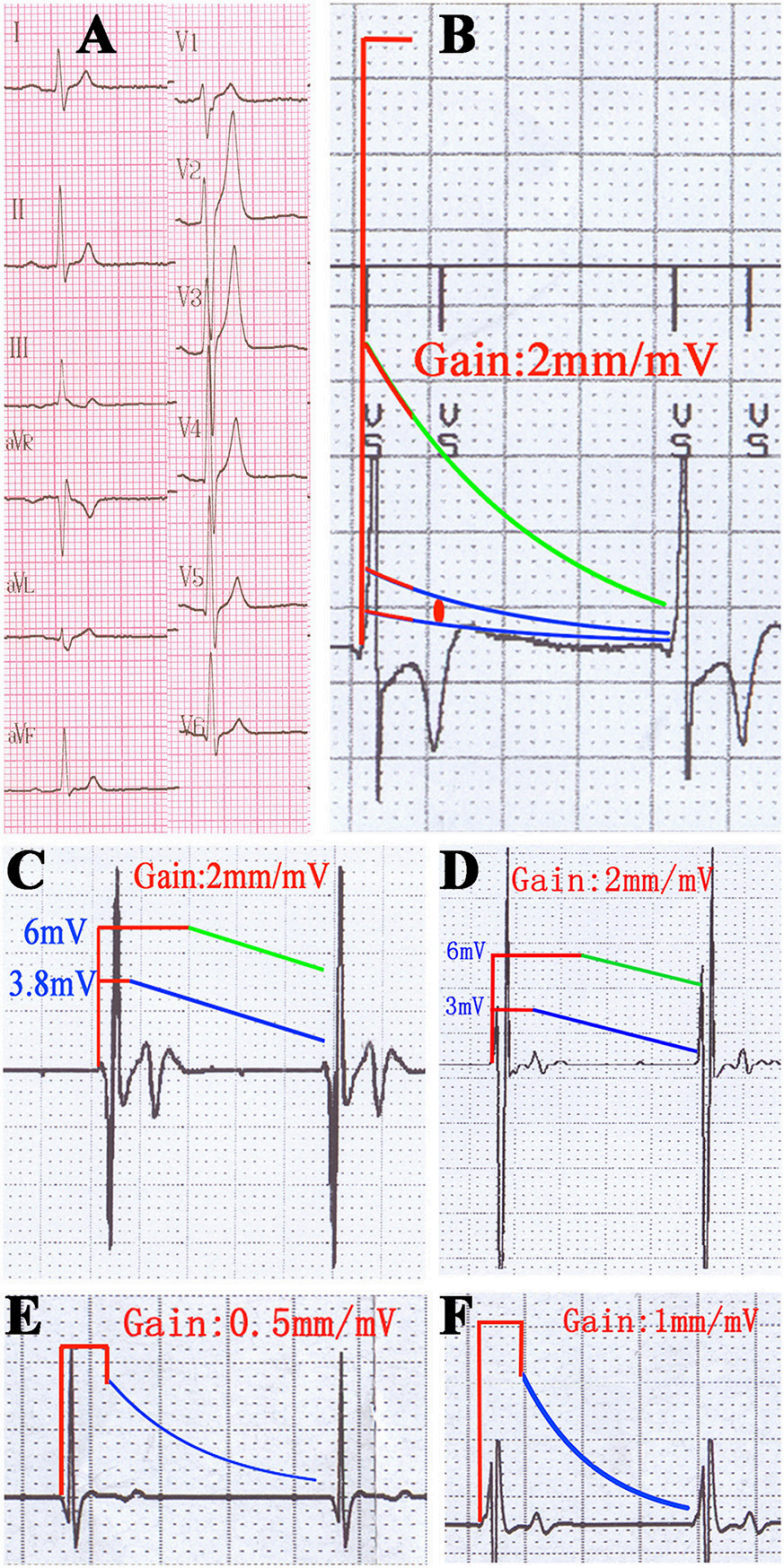
TWOS episodes during the procedure of ICD implantation and sensing threshold curves with real proportion. **(A)** 12-lead surface electrocardiogram of the short QT syndrome patient. **(B)** TWOS under the maximum sensitivity in Virtuoso ICD. Red line shows the blank period after sensing; green line shows the sensing curve with minimum sensitivity; blue lines show sensing curves with sensitivity of 0.3 and 0.15 mV; red dot shows the possible range of T wave amplitudes. **(C)** Intermittent TWOS under maximum sensitivity in Epic ICD. Green and blue lines show the sensing curves with minimum and default sensitivity, respectively. **(D)** Measurement of TWSM in Fortify ICD. **(E,F)** Measurement of TWSM in Vitality and Teligen ICDs. Blue lines show the sensing curve with default sensitivity.

In the TWOS test, nine patients were positive (28%). There were two sensing TWOS events and eight pacing TWOS events under the maximum sensitivity during the procedure of ICD implantation (Table 2). In the short QT syndrome patient, Virtuoso and Consulta ICDs showed TWOS at the maximum sensitivity of 0.15 mV at R-T interval of 200 ms (Figures 4A,B). No TWOS event was recorded at the sensitivity of 0.3 mV. The Epic ICD showed intermittent TWOS at the interval of 172 ms between R wave and T wave sensing at decay delay of 0 ms and threshold start of 50%. No TWOS event occurred at decay delay of 0 ms and threshold start of 62.5% (Figure 4C). Fortify, Vitality, and Teligen ICDs showed no sensing or pacing TWOS under maximum sensitivity (Figures 4D–F). The other sensing TWOS occurred in a patient with HCM, who also showed post pacing TWOS under the maximum sensitivity during the procedure of implantation.

**Table 2:** Details of patients with TWOS during the procedure of ICD implantation.

**‘Patient**	**Age, sex**	**Patho-genesis**	**Indication**	**Sensitivity**						**ICD implanted**	**Defibrillation lead**
				**0.15 mV**			**0.3 mV**				
				**VS**	**VP**	**PSVP**	**VS**	**VP**	**PSVP**		
1	54, M	SQTS	Primary	+	−	−	−	−	−	Vitality	Riata 1570
2	56, M	Brugada	Secondary	−	+	±	−	−	−	Fortify	Durata 7122
3	42, F	HCM	Primary	−	+	−	−	−	−	Fortify	Durata 7122
4	65, M	DCM	Primary	−	±	±	−	−	−	Current	Durata 7122
5	28, M	Brugada	Secondary	−	+	−	−	−	±	Teligen	RELIANCE G
6	52, F	SCD	Secondary	−	+	+	−	−	−	Fortify	Durata 7122
7	44, F	HCM	Primary	+	−	±	−	−	−	Teligen	RELIANCE G
8	58, M	Brugada	Primary	−	+	−	−	−	−	Current	Durata 7122
9	54, M	Post MI	Primary	−	+	+	−	+	−	Fortify	Durata 7122
10^*^	47, F	DCM	Primary	−	+ (BV)	−	−	−	−	Consulta	Sprint 6947

BV, Bioventricular pacing; ICD, implantable cardioverter-defibrillator; TWOS, T-wave oversensing; VP, ventricular pacing; VS, ventricular sensing; PSVP, post-sensing ventricular pacing. +, persistent TWOS; −, no TWOS; ±, intermittent TWOS;

* *during routine follow-up*.

**Figure 5:**
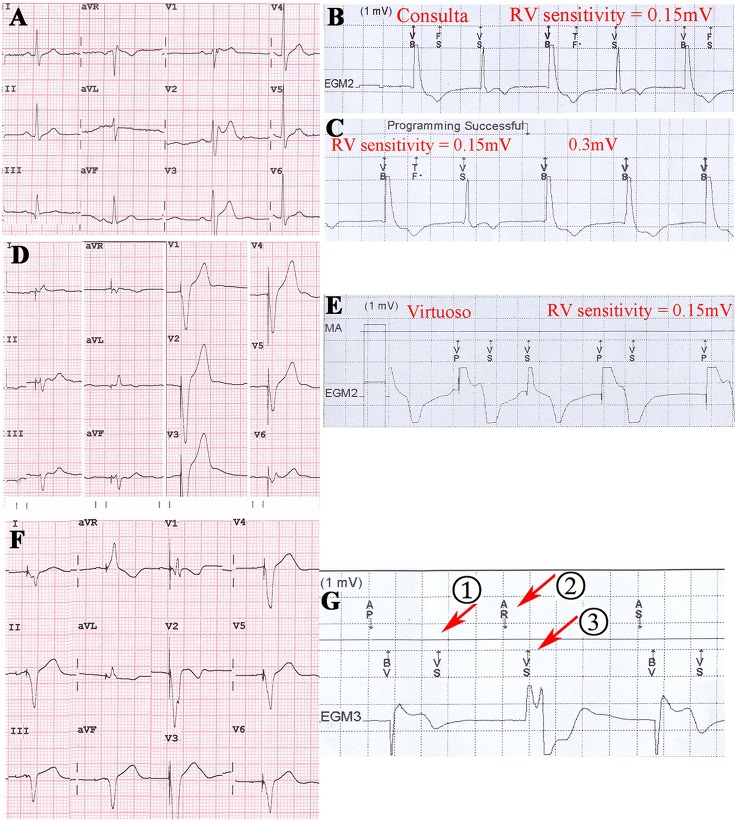
Post pacing TWOS revealed during the procedure of ICD implantation in Brugada syndrome patient, hypertrophic cardiomyopathy patient and dialated cardiomyopathy patient. **(A)** 12-lead surface EKG of a Brugada syndrome patient. **(B,C)** Post pacing TWOS episodes by a Consulta ICD under sensitivity of 0.15 mV. No TWOS was recorded under 0.3 mV. **(D)** 12-lead surface EKG of a hypertrophic cardiomyopathy patient. The implantable cardioverter-defibrillator working mode is VAT. **(E)** Post pacing TWOS episodes in Virtuoso ICD under the sensitivity of 0.15 mV. **(F)** 12-lead EKG of a CRT-D patient with dilated cardiomyopathy. **(G)** After the episode of TWOS (①), PVARP was prolonged by PVC response and sinus P wave was recognized as a premature beat (②) and not traced by ventricle (③).

Virtuoso and Consulta ICDs showed post pacing TWOS events in eight patients at a sensitivity of 0.15 mV, and in two patients under the maximum sensitivity of 0.3 mV (Figures 5A–E). No TWOS was recorded under the maximum sensitivity of 0.45 mV. The eight pacing TWOS patients included three with Brugada syndrome, two with hypertrophic cardiomyopathy, one with dilated cardiomyopathy, one who had survived cardiac arrest, and one post-myocardial infarction with low left ventricular ejection fraction. The two patients that had pacing TWOS events under the sensitivity of 0.3 mV were Brugada syndrome and hypertrophic cardiomyopathy, respectively.

### TWSM

With the default parameter setting, TWSMs post intrinsic rhythm sensing by Fortify and Teligen ICDs were 13.0 ± 7.6 and 28.3 ± 16.5, respectively (*n* = 32, Figure 3D). TWSM post sensing was not calculated in Epic, Virtuoso, Consulta or Vitality ICDs because T wave amplitudes were not measurable on these ICD printouts.

TWSMs post pacing by Fortify and Teligen ICDs were 5.0 ± 2.2 and 4.6 ± 0.9 respectively (*n* = 23, Figure 3D). TWSMs post sensing and pacing were not evaluated in Epic, Virtuoso, Consulta, or Vitality ICDs because T wave amplitudes were not measurable on these ICD printouts.

### Follow-up

The 32 patients were followed up for 25 ± 7 months. One DCM patients died of refractory heart failure. One Brugada patient was shocked three times for ventricular tachycardia and ventricular fibrillation. One short QT syndrome patient was shocked twice for fracture of defibrillation lead (Riata, St. Jude Medical, Inc.). Two patients experienced inappropriate ICD shocks for atrial fibrillation. One patient with a Consulta ICD showed TWOS after biventricular pacing in the default setting at the 3-month follow-up visit (Figures 5F,G, Table 2). No inappropriate therapy related to TWOS occurred during follow-up.

## Discussion

To the best of our knowledge, this is the first study that evaluates the T wave amplitude and TWSM of different ICDs during the procedure of ICD implantation. We first propose the index of TWSM and validated that ventricular signal amplitude could be measured on printouts in certain ICD models. *Ex vivo* connection could evaluate T wave amplitude and TWSM, and might reduce TWOS episodes at mid-term follow-up.

Currently, there is no reliable method to identify patients at high risk for TWOS. In clinical practice, if the R wave amplitude measured by the ICD programmer is high enough (>5~8 mV), the risk of TWOS is considered to be low. Recently, T/R ratio during AAI pacing using bipolar electrograms was reported as an excellent predictor of TWOS (Maesato et al., 2011). However, according to the sensing principle of ICDs, the devices sense the filtered ventricular signals when these signals are higher than the real-time sensing threshold. As T wave was concerned, it is TWSM rather than R wave amplitude or T/R ratio that determines the risk of TWOS when T wave amplitude varies. In this study, R wave amplitudes in all 32 patients were sufficiently high for clinical practice. But TWOS still occurred in some patients under the maximum sensitivity, which means insufficient TWSM in these patients under default parameters setting. In certain clinical situations, such as hyperkalemia, exercise, emotional fluctuation, and myocardial ischemia, T wave amplitude might increase and cause TWOS (Koul et al., 2004; Otmani et al., 2008). Evaluating T wave amplitude and TWSM during the procedure of ICD implantation might help clinicians to select optimal ICDs to reduce risk of TWOS by the following means: (1) When TWSM under default setting is <1 in one type of ICD, physician could implant another type with higher TWSM, or re-locate shock lead to avoid TWOS; (2) When TWOS occurs after ICD implantation and could not be solved by non-invasive method, replacing ICD generator with higher TWSM might resolve the problem and minimize the risk in the future; (3) When TWOS occurs during the follow-up period and could be solved by non-invasive method, TWSM could help to program appropriate sensing parameters.

To evaluate TWSM, two values are needed: T wave amplitude and real-time sensing threshold. The sensing threshold could be calculated according to the sensing algorithm of ICD (Supplementary Table 1). However, T wave amplitude could not be measured on an ICD analyzer or programmer, so we developed a novel method called *ex vivo* connection to measure T wave amplitude.

### *Ex vivo* connection

The intracardiac signal such as QRS wave is characterized by low amplitude (~mV) and high impedance (~kΩ). Therefore, modern ICD sensing circuitry needs high input impedance (~MΩ) to minimize the distortion of the QRS wave, so that the signal sampling is not significantly affected by trivial increases in load resistance (Myers et al., 1978). In this study, we evaluated R waves by *ex vivo* ICD connections, which produced similar patterns of ventricular sensing as implanted ICDs (data not shown). This suggests that ventricular sensing could be evaluated precisely by an *ex vivo* connection method before the ICD was implanted.

### R wave and T wave amplitude

In the sensing circuitry of ICD, both R and T waves are ventricular signals, so it is reasonable to measure R wave and T wave amplitude on ICD printouts. In our study, T wave amplitude post sensing was lower than after pacing in both Fortify and Teligen ICDs. This result was consistent with higher T waves after pacing than after sensing on surface EKGs. Interestingly, T wave amplitudes showed no difference between Fortify and Teligen ICDs, although the R wave amplitudes differed.

### TWOS and TWSM

In general clinical practice, ICD sensitivity is often programed to default values unless TWOS occurs. This “wait-and-see” strategy creates risks of TWOS and inappropriate therapy in ICD patients, especially in those at high risk of TWOS. In our study, 9/32 (28%) patients had episodes of TWOS at the maximum sensitivity settings. In these nine patients, default values could only provide a TWSM of <2 (i.e., 200%). When T wave amplitude increased for various clinical reasons, TWOS might occur. Sensing TWOS can cause inappropriate therapy, while pacing TWOS created a risk for inappropriate bradycardia and insufficient pacing in pacing-dependent patients, such as those with long QT syndrome and dilated cardiomyopathy (Iijima et al., 2011; Korantzopoulos et al., 2013). In our study, a dilated cardiomyopathy patient with a Consulta ICD showed TWOS post biventricular pacing during regular follow-up at 3 months. The TWOS resulted in a decreased pacing proportion. The situation was resolved by decreasing the ventricular sensitivity to 0.45 mV.

Further analysis showed that all nine episodes of TWOS occurred in the Epic ICDs and the two Medtronic ICDs. TWOS in the Epic ICD was caused by high T wave amplitude. It is noteworthy that the T wave amplitudes of the same patient were 2.4–3.8 mV by the Epic ICD, and 0.74 mV by the Fortify ICD, which might reflect technical improvement in the sensing circuitry of ICDs. In contrast, TWOS episodes in Medtronic ICDs were caused by low sensing thresholds rather than high T wave amplitudes. In Medtronic ICDs, sensing thresholds after sensing and pacing are limited to 8~10 and 4.5 times of the maximum sensitivity, respectively. At the default sensing threshold of 0.3 mV, the ventricular sensing thresholds are not more than 3 mV and 1.35 mV, respectively. This algorithm ensures high sensitivity to ventricular signals, but leads to a lower TWSM, especially in patients with high R wave amplitudes (Swerdlow and Friedman, 2005). Recently, a new TWOS rejection algorithm was employed in Medtronic ICDs, but its effectiveness in patients with a high risk of TWOS is still uncertain (Cao et al., 2012).

Subcutaneous ICDs (S-ICD), as an alternative to the transvenous ICD, are employed in recent years. The most common cause of inappropriate shocks in S-ICD was TWOS (Aydin et al., 2012; Olde Nordkamp et al., 2012; Kobe et al., 2013). Although, there are some methods to predict TWOS (Wilson et al., 2016) and resolve these problems (Kooiman et al., 2014), evaluation of TWSM during the procedure of ICD implantation seems to be a better choice to reduce TWOS in advance. However, the sensing signal in S-ICD is more similar to surface EKG rather than intracardiac signal in transvenous ICD. The validity of the hypothesis still needs further verification.

### Limitations

Only six ICD models are used to evaluate signal sensing. Some new types of ICD (e.g., Protecta and Evera of Medtronic and ICDs of Biotronik) are not tested because they are not available during our study. Even though paired comparisons employed in this study have adequate statistical power, 32 patients is a small sample population. T wave and TWSM were not fully evaluated under different heart rhythm or under different sympathetic active drugs (e.g., isoproterenol). Post-pacing T wave sensing is not fully tested for various reasons. Furthermore, R wave and T wave amplitudes may significantly change according to postural and emotional status, and should be further evaluated. The heterogeneity of the patients and lack of control group were the biggest drawback of our study. Further studies should be designed to clarify these issues.

## Conclusion

We first propose the index of TWSM during ICD implantation as a potentially efficient predictor for TWOS. Evaluation of TWSM may help to reduce TWOS episode in patients with high risk. Further studies are warranted to validate this predictor and its potential in clinical practice.

## Author contributions

DH, GF, YS, and HM. design the project. YS, JGao, XS, YY, and HH. coordinated and collected the clinical evaluations. DH, CJ, YMX, and YZX organized and summarized the data. YZ, GL, and JGuo. analyzed the data and made the figures and table. DH, YS, and JGao wrote the manuscript. All co-authors contributed to editing and proving of manuscript.

### Conflict of interest statement

The authors declare that the research was conducted in the absence of any commercial or financial relationships that could be construed as a potential conflict of interest.
