# Supplementary material for: T Wave Safety Margin during the Process of ICD Implantation As a Novel Predictor of T Wave Oversensing
Source: Front Physiol. 2017 Sep 1;8:659. doi: 10.3389/fphys.2017.00659 (PMC5585188; doi:10.3389/fphys.2017.00659)
Supplement: Supplementary file 1 [file Table1.docx]

**T-wave Safety Margin during the Process of ICD Implantation as a Novel Predictor of T Wave Oversensing**

*Short title: Sun, et al - T-wave Safety Margin* *as a Novel Predictor*

YA-XUN SUN, M.D., Ph.D.* JING GAO, M.D.,* CHEN-YANG JIANG, M.D., YU-MEI XUE, M.D., YI-ZHOU XU, M.D., GANG LIU, M.D., JI-HONG GUO, M.D.,^­­^ XIA SHENG, M.D., YANG YE, M.D., HONG HE, M.D., YUN-TAO ZHAO, M.D., HECTOR BARAJAS-MARTINEZ, Ph.D., GUO-SHENG FU, M.D., Ph.D.,^#^ DAN HU, M.D., Ph.D. ^#^

*The first two authors contributed equally to this work.

From the Department of Cardiology, Sir Run Run Shaw Hospital, School of Medicine, Zhejiang University, Hangzhou, 310016, China (Y.S., J.G.[Gao], C.J., X.S., Y.Y., H.H., G.F.);

Department of Cardiology, Guangdong Cardiovascular Institute, Guangdong General Hospital, Guangzhou, 510080, China (Y.X.);

Department of Cardiology, Hangzhou First People's Hospital, Hangzhou, 310006, China (Y.X.);

Department of Cardiology, The first affiliated hospital of Hebei Medical University, Shijiazhuang, 050023, China (G.L.);

Division of Cardiology, People's Hospital, Peking University, Beijing, 100044, China (J.G.[Guo]);

Department of Cardiology, Aerospace Center hospital, Beijing, 1000766, China (Y.Z.);

Department of Cardiology and Cardiovascular Research Institute, Renmin Hospital of Wuhan University, Wuhan, 430060, China (D.H.);

Masonic Medical Research Laboratory, Utica, New York, 13501 (D.H., H.B.M.).

Correspondence to:

| **Guo-Sheng Fu**, M.D. Ph.D., FACC, FESC, FSCAI  Director of Cardiology Department  Sir Run Run Shaw Hospital,  School of Medicine,  Zhejiang University,  HangZhou, 310016, China.  Phone: 86-571-86006496  FAX: 86-571-86006246  E-mail: [fugs@zju.edu.cn](mailto:fugs@zju.edu.cn) | **Dan Hu**, MD. PhD. FAHA. FACC. FHRS.  Research Scientist II, Associate Professor  Clinical Consultant of Molecular Genetic Department  SCRO Chair of Stem Cell Center  Masonic Medical Research Laboratory  2150 Bleecker St, Utica, NY 13501  Phone: (315) 624-7482  FAX: (315) 735-5648  Email: [dianah@mmrl.edu](mailto:dianah@mmrl.edu) |
| --- | --- |

**Journal Subject Term:**  **Arrhythmia and Electrophysiology - Catheter Ablation and Implantable Cardioverter-Defibrillator**

**Supplementary Table 1. Algorithms and parameters of six testing ICDs**

| **Model** | | **St. Jude＊** | | | **Boston Scientific #** | | **Medtronic†** | |
| --- | --- | --- | --- | --- | --- | --- | --- | --- |
|  |  | **Epic**  **V239** | **Fortify DR2231-40** | | **Vitality**  **F167** | **Teligen**  **F110** | **Virtuoso**  **VR D164VWC** | **Consulta**  **D234TRK** |
| Type | | Dual-C ICD | Dual-C ICD | | Dual-C ICD | Dual-C ICD | Single-C ICD | CRT-D |
| Testing lead | | Guidant Fineline II EZ 4471 | | | | | | |
| Maximum sensed ventricular amplitude | | 12 mV | | | 24 mV | 32 mV | 20 mV | 20 mV |
| Post sensing blank period  (default) | | 125 + 60 ms | | | 135 ms | 135 + 15 ms | 120 ms | |
| Sensing threshold | | 50, 62.5, 75, and 100% | | | 75% | | 75% | |
| Sensing algorithm | Sense | S = T_s_*R_s_ - (t- t­_R_-t_dd_)/312 | | | S = 0.75R_s_*(1/2)^[(t-135)/200]  (Sinus rhythm)  S=0.75R_s_*(1/2)^[(t-135)/150] (Tachycardia)  (Vitality) | | S = 0.75*R_s_* (1/3)^t/450^  R_s_ = R  (0.75*R ≤ 8*S_max_);  R_s_ = 8S_max_/0.75  (0.75*R > 8*S_max_) | |
|  |  |  |  |  | S = 0.75R_s_*(7/8)^[(t-135-15)/35]  (Teligen) | |  |  |
|  | Pace | Automatically | | | Adjusted automatically according to pacing rate. Reached maximum sensitivity 200 ms before next pacing.  s = 8*0.75*0.6*(7/8)^[(t-135-15)/( (60000/90-150-135-15)/ (ln(1/6)/ln(7/8)))]  (90 ppm) | | S = 4.5*S_max_* (1/3)^t/450^;  (S_max_ ≤ 0.4 mV)  S = 1.8* (1/3)^t/450^;  (S_max_ > 0.4 mV）  Blanking period: 60ms | |
| R-wave amplitude and R-wave for algorithm | | R_s_ = 6 mV; (R > 6mV)  R_s_ = R; (2 mV ≤ R ≤6 mV)  Rs = 2 mV; (R < 2 mV) | | | R_s_= 24 mV  (R > 24 mV);  R_s_ = R  (R < 24 mV) | R_s_ = 32 mV  (R > 32 mV);  R_s_ = R  (R < 32 mV) | R_s_ = R(0.75*R ≤ 8*S_max_);  R_s_ = 8S_max_/0.75 (0.75*R > 8*S_max_) | |
| Default sensitivity | | 60 ms 62.5% | 60 ms 50% | | AGC 0.27 mV | AGC: 0.6 mV | S_max_: 0.3 mV | |
| Maximum sensitivity | | 0ms, 50% | | | 0.15mV | Most | S_max_: 0.15 mV | |
| Blank ventricular period | | 125 ms | | | 135 ms | | 120 ms | |
| Ventricular channel | | Bipolar | | Bipolar/  Amplifier | Ventricular | | Bipolar | |
| Measured R amplitude on IEGM consistent with R amplitude by ICD | | No | | Yes  (Amplifier) | Yes | | No | |

Abbreviations: ICD, implantable cardioverter-defibrillator; R, ventricular amplitude; R_s_, ventricular amplitude for ICD sensing algorithm; S_max_, maximum sensitivity threshold; t , interval from R-sense to T-sense; t_dd_, decay delay; t_R_, refractory time; T_s_, threshold start.

*****The Threshold Start of EPIC V239 and Fortify can be programmed over the range of 50%, 62.5%, 75% and 100%. At nominal settings, the default T_s_ begins at 62.5% (EPIC V239) and 50% (Fortify DR2231-40) of the sensed R wave. The amplitude of the sensed R-wave is equal to that of the measured R-wave only when its value is between 2–6 mV. If the measured R-wave amplitude is > 6 or < 2 mV, the maximum and minimum sensed R-wave amplitude are 6 and 2 mV, respectively. At maximum sensitivity, the value of t_dd_ is 0 ms and the threshold is set to 50% of the sensed R-wave amplitude.

**†** The formulas of the Virtuoso VRD164VWC and Consulta D234TRK algorithms after a paced R wave is 4.5*S_max_* (1/3)^t/450^ if the value of S_max_ is ≤ 0.4 mV or 1.8* (1/3)^t/450^ if the value of S_max_ is > 0.4 mV. After a sensed R wave, the algorithm is 0.75*R_s_* (1/3)^t/450^. The amplitude of R_s_ is equal to that of the measured R wave when its value is ≤ 8*S_max_ or 8*S_max_/0.75 if 75% of the amplitude of the measured R wave is > 8*S_max_. The value of default sensitivity and maximum sensitivity is 0.3 and 0.15 mV, respectively.

**#** Vitality F167 and Teligen F110 set the starting threshold at 75% of the sensed R wave. Under the sinus rhythm and after a sensed R wave, the algorithm of Vitality F167 is 0.75R_s_*(1/2)^[(t-135)/200], but if tachycardia is sensed, the sense algorithm is 0.75R_s_*(1/2)^[(t-135)/150]. The algorithm of Teligen F110 is 0.75R_s_*(7/8)^[(t-150)/35]. The amplitude of the sensed R wave equals that of the measured R wave when its value is < 24 mV (Vitality F167) or < 32 mV (Teligen F110). However, if the measured R-wave amplitude is > 24 or > 32 mV, the maximum sensed R-wave amplitude of Vitality F167 and Teligen F110 is 24 and 32 mV, respectively. The default sensitivity (AGC) of Vitality F167 is 0.27 mV and that of Teligen F110 is 0.6 mV. To reach maximum sensitivity, the value of AGC can be set to 0.15 mV (Vitality F167) or the most sensitive status (Teligen F110).
